# Longitudinal change in estimated GFR among CKD patients: A 10-year follow-up study of an integrated kidney disease care program in Taiwan

**DOI:** 10.1371/journal.pone.0173843

**Published:** 2017-04-05

**Authors:** Ching-Wei Tsai, I-Wen Ting, Hung-Chieh Yeh, Chin-Chi Kuo

**Affiliations:** 1 Division of Nephrology and Kidney Institute, Department of Internal Medicine, China Medical University Hospital, Taichung, Taiwan; 2 School of Medicine, College of Medicine, China Medical University, Taichung, Taiwan; 3 Big Data Center, China Medical University Hospital, Taichung, Taiwan; The University of Tokyo, JAPAN

## Abstract

**Background:**

This study examined the progression of chronic kidney disease (CKD) by using average annual decline in estimated GFR (eGFR) and its risk factors in a 10-year follow-up CKD cohort.

**Methods:**

A prospective, observational cohort study, 4600 individuals fulfilled the definition of CKD, with or without proteinuria, were followed for 10 years. The eGFR was estimated by the MDRD equation. Linear regression was used to estimate participants’ annual decline rate in eGFR. We defined subjects with annual eGFR decline rate <1 ml/min/1.73 m^2^ as non-progression and the decline rate over 3 ml/min/1.73 m^2^ as rapid progression.

**Results:**

During the follow-up period, 2870 (62.4%) individuals had annual eGFR decline rate greater than 1 ml/min/1.73 m^2^. The eGFR decline rate was slower in individuals with CKD diagnosed over the age of 60 years than those with onset at a younger age. Comparing to subjects with decline rate <1 ml/min/1.73 m^2^/year, the odds ratio (OR) of developing rapid CKD progression for diabetes, proteinuria and late onset of CKD was 1.72 (95% CI: 1.48–2.00), 1.89(1.63–2.20) and 0.68 (0.56–0.81), respectively. When the model was adjusted for the latest CKD stage, comparing to those with CKD stage 1, patients with stage 4 and stage 5 have significantly higher risks for rapid progression (OR, 5.17 (2.60–10.25), 19.83 (10.05–39.10), respectively). However, such risk was not observed among patients with the latest CKD stage 2 and 3. The risk for incident ESRD was 17% higher for each 1 ml/min/1.73 m^2^ increasing in annual decline rate.

**Conclusions:**

Not everyone with CKD develops ESRD after a 10-year follow-up. Absolute annual eGFR decline rate can help clinicians to better predict the progression of CKD. Individuals with renal function decline rate over 3 ml/min/1.73 m^2^/year require intensive CKD care.

## Introduction

Worldwide, the continuously increasing prevalence of chronic kidney disease (CKD) becomes a major public health concern. CKD patients frequently progress to end-stage renal disease (ESRD) requiring a renal-replacement therapy (RRT), which incurs a substantial healthcare cost burden [1]. Taiwan is the country holding the highest healthcare burden of ESRD in the world with an estimated incidence and prevalence of 458 per million and 3,138 per million, respectively, compared with 363 and 2,043, respectively, of the US in 2013 [2–4]. CKD patients also have much higher risk for cardiovascular disease (CVD) events and mortality than general population, further straining the healthcare system (2). It is therefore imperative to identify predictors for CKD progression.

Current CKD guidelines suggest a stage-based approach to standardize the disease severity and design corresponding therapeutic plans [5, 6]. The widely used CKD staging system is based on the level of creatinine-based estimated GFR (eGFR) and the magnitude of proteinuria [5, 6]. However, the rate of progression in eGFR may vary irrespective of the baseline GFR [7–9]. This helps explain why patients with similar baseline CKD stage may have different disease course and outcome along the course of CKD. The rate of progression in eGFR may serve as a more relevant indicator to quantify disease severity and a useful domain for monitoring treatment response [10]. A recent meta-analysis showed that the declining rate in eGFR is independently associated with the risk of ESRD and mortality after adjustment for baseline eGFR[11]. In this study, we take the advantage of using a prospective cohort with a 10-year follow-up to evaluate the association between annual decline rate of eGFR and the risk of developing ESRD and to identify the factors associated with a rapid eGFR decline rate.

## Methods

### Ethics statement

The study has been approved by the Big Data Center of China Medical University Hospital and the Research Ethical Committee/Institutional Review Board of China Medical University (CMUH105-REC3-068). Informed consent was not obtained from the study participants because the data was analyzed anonymously and was in accordance with Institutional Review Board guidelines. The Institutional Review Board has verified the anonymity of data analysis performed in this study.

### Study population

Taiwan's National Health Insurance (NHI) has launched the Project of Integrated Care of CKD since 2002 [12]. China Medical University Hospital (CMUH), a tertiary medical center in Central Taiwan, joined this program since 2003 and prospectively enrolled patients with CKD stages 1 to 5 who were willing to participate [13]. The diagnosis of CKD was according to the criteria of the National Kidney Foundation Kidney Disease Outcomes Quality Initiative (NKF/KDOQI) Clinical Practice Guidelines for CKD [14]. Patients in this program were regularly followed up at outpatient department. Biochemical markers of renal injury including serum creatinine, blood urea nitrogen, as well as the spot urine protein to creatinine ratio (UPCR) were measured at least every 12 weeks or more frequent. All enrolled patients were followed-up until initiation of long-term renal replacement therapy (hemodialysis, peritoneal dialysis, or transplantation), loss to follow-up, death, or December 31, 2013, whichever occurred first. To avoid the deviation of eGFR trajectory due to episodes of acute kidney injury, we did not include laboratory data measured during a hospital admission. In the final analysis, we included 4,600 patients who had joined this integrated care program of CKD at CMUH aged 18 years or above with at least three outpatient creatinine measurements over a period of at least 12 months staying within the program between 2004–2013.

### Measurement of kidney function

The eGFR was estimated using the abbreviated Modification of Diet in Renal Disease (MDRD) equation (eGFR = 175 x creatinine^-1.154^ x age^-0.203^ x 1.212 [if black] x 0.742 [if female])[15]. The serum creatinine level at enrollment was used to define the baseline eGFR and corresponding CKD stage using the cut-off values: >90, 60–89.9, 30–59.9, 15–29.9, and <15 ml/min/1.73 m^2^. Of all prospective outpatient eGFR measurements within the included subjects until the development of end-points, the latest eGFR available before the end-point was used to define the latest CKD stage. The average annual decline of eGFR was estimated by the difference between baseline eGFR and the latest available eGFR divided by time interval in years. We defined subjects with annual eGFR decline rate over 1 ml/min/1.73 m^2^ as moderate progression and decline rate over 3 ml/min/1.73 m^2^ as rapid progression [16, 17]. Outpatient random spot urine dipstick and UPCR measurements were used to estimate proteinuria. Proteinuria was defined as in a least two of three consecutive urine exam showing urine dipstick> 1+ or proteinuria >0.5 g/day from random spot urine. The quantification of proteinuria was determined at the same time as the serum creatinine concentration was measured.

### Other variables

Diabetes was defined as having a fasting glucose level higher or equal than 126 mg/dL, or a non-fasting glucose level of 200 mg/dL or higher, hemoglobin A1c level of 6.5% or higher, use of glucose-lowering drugs, or self-reported diabetes. Diabetic nephropathy was either confirmed by renal biopsy or clinical diagnosis by nephrologists. History of cardio-vascular disease (CVD) is defined as documented coronary artery disease, myocardial infarction, stroke and heart failure in the electronical medical records.

### Statistical analyses

Continuous variables were expressed as means ± standard deviation (SD) and categorical variables were expressed by percentages. Multiple linear regression analysis was performed to evaluate the associations between annual eGFR declines with baseline co-variables. The co-variables included age, sex, proteinuria (yes vs. no), hypertension (yes vs. no), diabetic nephropathy (yes vs. no), history of CVD, and baseline CKD stage. A multiple logistic regression analysis was performed to determinate the factors associated with rapid eGFR decliners (annual decline rate >3 ml/min/m^2^). We also investigated the association between the annual eGFR declining rate with the risk of incident ESRD using multivariable cox proportional hazards modeling. Models were adjusted for age, sex, BMI, smoking, hypertension, diabetes, CVD, hyperlipidemia, proteinuria and baseline eGFR. Kaplan-Meier analysis was used to plot kidney survival curve.

We used age as the time scale and the follow-up time in the Cox proportional hazards analysis was the time between the baseline eGFR measurement and the initiation of dialysis. Mortality, kidney transplantation, and loss to follow up were treated as censored events. All analyses were conducted using STATA statistical software, version 12.0 (StataCorp LP, College Station, Texas).

## Results

### Overview of the study population

During a total of 10,314 person-years of follow-up (the mean follow-up time was 2.24 years), 2870 (62.4%) individuals had annual eGFR decline rate greater than 1 ml/min/1.73 m^2^ per year. The median average annual eGFR decline rate was 4.42 and -1.98 ml/min/1.73 m^2^ per year for those renal functions classified as progression and non-progression, respectively. Baseline characteristics of two groups are listed in Table 1. Patients with annual decline rate ≥ 1 ml/min/1.73 m^2^ per year were significantly younger, more likely to be female, and with higher BMI, systolic blood pressure (SBP), and diastolic blood pressure (DBP) at the enrollment of pre-ESRD program compared to those with <1 ml/min/1.73 m^2^ per year and they had a higher average baseline serum creatinine level with a corresponding lower baseline eGFR. The prevalence of hypertension, diabetes and proteinuria were all higher among those having faster eGFR declining rate (Table 1). In the non-progression group, most patients were classified as having CKD stage 3 at baseline and the combined proportion of baseline stage 1–3 was 51.2%, while in the progression group, 64.8% patients had CKD stage 4 or 5 at baseline. At the end of follow-up, we found that the combined proportion the latest stage 1–3 among non-progression patients was increased to 63.0% while only 18.5% of progression group remained at the CKD stage 1–3.

**Table 1:** Baseline characteristics of patients with chronic kidney disease stratified by eGFR decline rate during a 10-year follow-up period.

Variables	Total	Non-progression eGFR-ADR<1 ml/min/1.73m^2^	Progression GFR-ADR≥1 ml/min/1.73m^2^	*p*-value
N	4600	1730	2870	
***Demographics and Cormobidities***				
Age (year)	70.1(0.20)	71.3 (0.33)	69.4 (0.26)	<0.001
Male (%)	2640 (57.4%)	1068 (61.7%)	1572 (54.8%)	<0.001
Body mass index (kg/m^2^)	25.5 (0.6)	24.6 (1.0)	26.0 (0.8)	<0.001
Smoking (%)	447 (9.7%)	166 (9.6%)	281 (9.8%)	0.8
End stage renal disease(%)	1328 (28.9%)	171 (9.9%)	1157 (40.3%)	<0.001
Hypertension (%)	3682 (80.0%)	1351 (78.1%)	2331 (81.2%)	0.01
Cardiovascular disease(%)	506 (11.0%)	204 (11.8%)	302 (10.5%)	0.2
Dyslipidemia (%)	397 (8.6%)	142 (8.2%)	255 (8.9%)	0.4
Diabetes (%)	1971 (42.9%)	627 (36.2%)	1344 (46.8%)	<0.001
Diabetic nephropathy (%)	1765 (38.4%)	535 (30.9%)	1230 (42.9%)	<0.001
Systolic blood pressure (mmHg)	135.2 (0.28)	131.8 (0.44)	137.3 (0.35)	<0.001
Diastolic blood pressure (mmHg)	77.2 (0.18)	76.6 (0.29)	77.6 (0.22)	0.004
Mean arterial pressure (mmHg)	96.6 (0.17)	95.0 (0.29)	97.5 (0.23)	<0.001
***Biochemical profile***				
BUN (mg/dL)	45.5 (0.57)	43.6 (0.95)	46.6 (0.71)	0.013
Creatinine (mg/dL)	3.17 (0.04)	2.97 (0.06)	3.29(0.04)	<0.001
Proteinuria (%)	2651 (57.6%)	829 (47.9%)	1822 (63.5%)	<0.001
Baseline eGFR (ml/min/1.73m^2^)	29.3 (0.31)	32.4 (0.49)	27.4 (0.38)	<0.001
Median eGFR-ADR (ml/min/1.73m^2^/year)	2.11	-1.98	4.42	<0.001
***Baseline CKD stage***				
Stage 1–2 (%)	310 (6.74%)	120 (6.94%)	190 (6.62%)	<0.001
Stage 3(%)	1586 (34.48%)	765 (44.22%)	821 (28.61%)	
Stage 4(%)	1341 (29.15%)	468 (27.05%)	873 (30.42%)	
Stage 5(%)	1363(29.63%)	377 (21.79%)	986 (34.36%)	
***Latest CKD stage***				
Stage 1–2 (%)	397 (8.63%)	291 (16.82%)	106 (3.69%)	<0.001
Stage 3(%)	1224 (26.61%)	798 (46.13%)	426 (14.84%)	
Stage 4(%)	852 (18.52%)	332 (19.19%)	520 (18.12%)	
Stage 5(%)	2127 (46.24%)	309 (17.86%)	1818 (63.34%)	

**Abbreviations:** eGFR-ADR: estimated glomerular filtration rate- annual decline rate

#### Subgroup analysis

**Fig 1:**
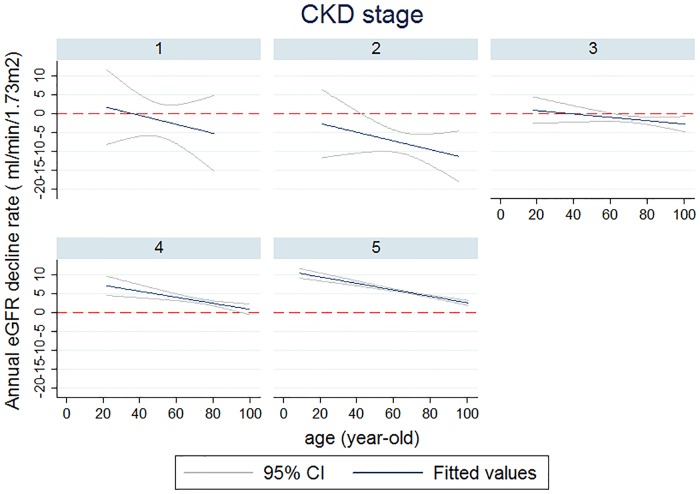
Average annual eGFR decline rate by age and CKD stage.

When we stratified patients by conventional risk factors of CKD, there were significant differences in eGFR decline rate between subgroups (Table 2). The annual eGFR decline rate was 3.96 ± 0.30 ml/min/1.73m^2^ for patients with diabetic nephropathy. CKD patients with proteinuria had significantly faster renal function decline than those without (3.28± 0.25 v.s. 0.36± 0.29 ml/min/1.73m^2^, p<0.001). However, the annual decline rate in eGFR did not differ by gender and between those with and without CVD or obesity. The average annual decline rate of eGFR was slower among individuals with CKD diagnosed after the age of 60 years than those diagnosed before the age of 60 years (Table 2). Fig 1 showed average annual eGFR decline rate was less steep with increasing age in CKD stage 3, 4 and 5. Also, the annual eGFR decline rate was > 0 ml/min/1.73m^2^ only in CKD stage 4 and 5 (Fig 1).

**Table 2:** The eGFR annual decline rate by subgroups.

Subgroup	Reference group	N	Annual eGFR decline (ml/min1.73 m^2^ per year)	*p*-value	Age-adjusted annual eGFR decline (ml/min1.73 m^2^ per year)	*p*-value
**Age**	Age<60 years	1010	3.11 (0.41)	0.003	4.20 (0.59)	0.9
	Age≥60 years	3619	1.75 (0.21)		4.25 (1.00)	
**Sex**	Female	1960	2.18 (0.29)	0.51	4.37 (0.62)	0.45
	Male	2640	1.94 (0.25)		4.08 (0.60)	
**DMN**	No	2835	0.85 (0.24)	<0.001	3.14 (0.59)	<0.001
	Yes	1765	3.96 (0.30)		6.31 (0.63)	
**Hypertension**	No	918	1.51 (0.42)	0.16	3.62 (0.67)	0.094
	Yes	3682	2.17 (0.21)		4.41 (0.59)	
**CVD**	No	4094	2.02 (0.20)	0.8	4.19 (0.58)	0.5
	Yes	506	2.19 (0.57)		4.61 (0.83)	
**Proteinuria**	No	1953	0.36 (0.29)	<0.001	2.01 (0.65)	<0.001
	Yes	2676	3.28 (0.25)		4.77 (0.58)	
**Dyslipidemia**	No	4203	2.05 (0.67)	0.9	4.24 (0.59)	0.7
	Yes	397	2.00 (0.64)		3.95 (0.81)	

**Abbreviations:** DMN: Diabetic Nephropathy; CVD: Cardiovascular disease

#### Risk factors associated with annual decline rate of eGFR and progression status

Compared to individuals without diabetes or proteinuria, multiple linear regression analyses revealed that diabetes, proteinuria and mean arterial pressure significantly associated with an increased annual decline rate of 1.63 (95% confident interval (C.I.): 0.88–2.38), 2.38 (1.62–3.14) ml/min/1.73 m^2^ and 0.08 (0.04–0.11), respectively (Table 3). In contrast, gender, dyslipidemia, or CVD history did not associate with the eGFR decline rate. We defined subjects with annual eGFR decline rate over 3 ml/min/1.73 m^2^ as rapid progression. In multiple logistic regression with adjustment for baseline eGFR, comparing to subjects with decline rate <1 ml/min/1.73 m^2^, the odds ratio of diabetes, proteinuria and age of CKD diagnosed over 60 years, for developing rapid progression was 1.72 (95% CI: 1.48–2.00), 1.89(1.63–2.20) and 0.68 (0.56–0.81), respectively (Table 4, model 1). When the model was adjusted for baseline CKD stage, neither early nor late CKD stages were associated with rapid progression (Table 4, model 2). When the model was adjusted for the latest CKD stage, comparing to those with the latest CKD stage 1, patients with the latest CKD stage 4 and 5 have significantly higher risks for developing rapid progression (odds ratio, 5.17 (95% CI: 2.60–10.25), 19.83 (95% CI: 10.05–39.10), respectively) (Table 4, model 3).

**Table 3:** Regression coefficients between the eGFR annual decline rate and the common risk factors in the multiple linear regression modeling.

Variable	β coefficient (95% CI)	*p*-value
**Age (per year)**	- 0.024 (-0.052, 0.004)	0.098
**Sex (ref: female)**	-0.67 (-1.43, 0.09)	0.08
**Smoking (ref: No)**	0.28 (-0.94, 1.51)	0.65
**Diabetes (ref: No)**	1.63 (0.88, 2.38)	<0.001
**MAP (mmHg)**	0.08 (0.04, 0.11)	<0.001
**Proteinuria (ref: No)**	2.38 (1.62, 3.14)	<0.001
**dyslipidemia (ref: No)**	-0.94 (-2.24, 0.36)	0.2
**CVD (ref: No)**	0.49 (-0.65, 1.64)	0.4
**Baseline eGFR (per 1 ml/min/1.73m^2^)**	0.04 (0.03, 0.06)	<0.001

**Abbreviations:** DMN: Diabetic Nephropathy; CVD: Cardiovascular disease; MAP: mean arterial pressure

**Table 4:** The odds ratio for rapid CKD progression [rapid progression (eGFR-ADR >3 ml/min/1.73m^2^) vs. non-progression (eGFR-ADR <1 ml/min/1.73m^2^)] of different matrices of kidney function in multiple logistic regression.

Kidney function	Model 1	Model 2	Model 3
	Baseline eGFR	Baseline CKD stage	Latest CKD stage
**per 1 ml/min/1.73m**^**2**^	0.995 (0.991, 0.999)**	—	—
**Stage 1**	—	Ref	Ref
**Stage 2**	—	1.02 (0.56, 1.88)	1.04 (0.50, 2.14)
**Stage 3**	—	0.86 (0.50, 1.49)	1.90 (0.96, 3.74)
**Stage 4**	—	1.33 (0.77, 2.30)	5.17 (2.60, 10.25)***
**Stage 5**	—	1.26 (0.73, 2.18)	19.83 (10.05, 39.10)***
**Age > 60 years (ref: ≤ 60)**	0.68 (0.56, 0.81) ***	0.68 (0.57, 0.82)***	0.55 (0.44, 0.68) ***
**Sex (Ref: female)**	0.84 (0.72, 0.98)*	0.87 (0.75, 1.02)	1.09 (0.92, 1.30)
**Diabetes (Ref: no)**	1.72 (1.48, 2.00) ***	1.69 (1.46, 1.97)***	1.64 (1.38, 1.95) ***
**MAP (mmHg)**	1.02 (1.01, 1.03)***	1.02 (1.01, 1.03)***	1.01 (0.999, 1.014)***
**Proteinuria (Ref: no)**	1.89 (1.63, 2.20) ***	1.83 (1.57, 2.13)***	1.47 (1.23, 1.74) ***
**Dyslipidemia (Ref: no)**	1.10 (0.85, 1.43)	1.12 (0.86, 1.45)	1.29(0.95, 1.75)
**CVD (Ref: no)**	1.07 (0.86, 1.33)	1.07 (0.85, 1.33)	1.02 (0.79, 1.31)
**Smoking (Ref: no)**	1.12 (0.88, 1.43)	1.11 (0.87, 1.42)	1.11 (0.85, 1.46)

Significance:

* *p*-value<0.05;

** *p*-value <0.01;

*** *p*-value<0.001

Abbreviations: CKD, chronic kidney disease; eGFR-ADR: estimated glomerular filtration rate—annual decline rate. CVD, cardiovascular disease; ADR: annual decline rate; MAP: mean arterial pressure

#### Annual declining rate and the risk of developing ESRD

The risk for incident ESRD was 17% (95% CI 1.16–1.18) higher for each 1 ml/min/1.73 m^2^ increase in annual decline rate after adjustment for diabetes, age at CKD diagnosis, proteinuria and baseline CKD stage (Table 5, model 2).

**Table 5:** The hazard ratio for the risk of end stage renal disease of eGFR-ADR and other common risk factors according to multiple Cox regression proportional hazard analysis.

	Model 1	Model 2
Variable	Hazard ratio (95%CI)	Hazard ratio (95% CI)
**Annual decline rate (per 1 ml/min/1.73m^2^)**	1.12 (1.12, 1.13)***	1.17 (1.16, 1.18)***
**Age > 60 years (ref: ≤ 60)**	0.19 (0.12, 0.31)***	0.55 (0.48, 0.64)***
**Sex (Ref: female)**	1.28 (1.14, 1.44)***	1.46 (1.28, 1.67)***
**Diabetes (Ref: no)**	1.45 (1.29, 1.63)***	1.88 (1.65, 2.14)***
**Proteinuria (Ref: no)**	1.24 (1.09, 1.38)***	1.10 (0.96, 1.23)
**Dyslipidemia (Ref: no)**	1.12 (0.91, 1.18)	1.12 (0.91, 1.18)
**CVD (Ref: no)**	1.11 (0.91, 1.34)	1.27 (1.03, 1.56)
**Smoking (Ref: no)**	1.04 (0.86, 1.26)	1.18 (0.98, 1.45)
**Baseline eGFR (per 1 ml/min/1.73m^2^)**	0.88 (0.87, 0.88)***	0.85 (0.84, 0.86)***
**Hypertension (Ref: no)**	1.07 (0.92, 1.25)	
**MAP(mmHg)**		1.02 (1.01, 1.02)***

Significance:

* *p*-value<0.05;

** *p*-value <0.01;

*** *p*-value<0.001

Abbreviations: CVD, cardiovascular disease; eGFR-ADR: estimated glomerular filtration rate—annual decline rate; MAP: mean arterial pressure

**Fig 2:**
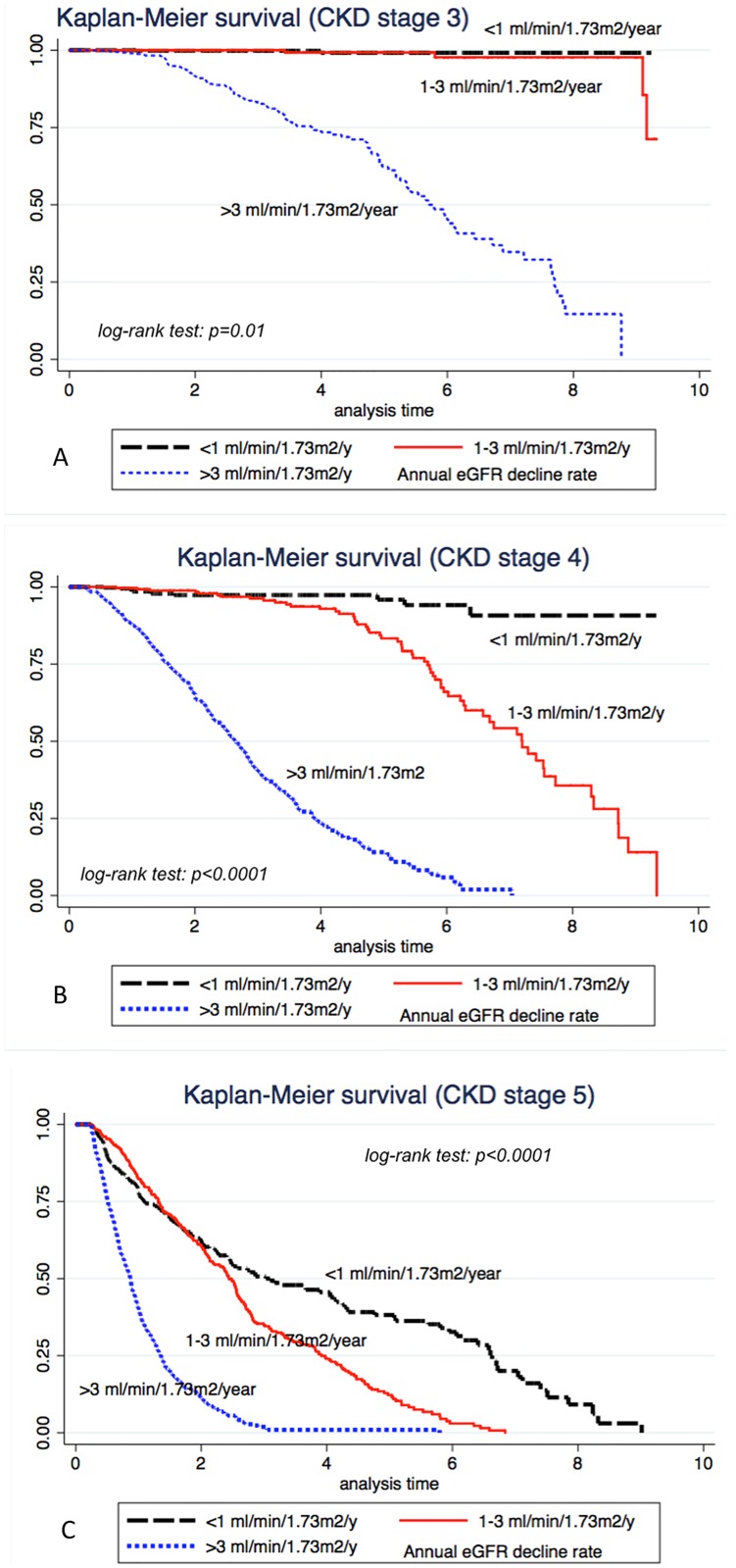
The survival curve before enter ESRD in individuals diagnosed at (A) stage 3, (B) stage 4, (C) stage 5, stratified by average annual decline rate.

In patients with initial CKD stage 3, 50% of them with rapid progression (annual decline rate > 3 ml/min/1.73m^2^) entered into ESRD within 5 years based on crude Kaplan-Meier survival curve (Fig 2A). In contrast, for individuals with initial CKD stage 3 and annual decline rate 1–3 ml/min/1.73m^2^ and those with <1 ml/min/1.73m^2^, the renal survival did not differ in the first 5 years after diagnosis. For those individuals with annual decline rate < 1 ml/min, although they had been classified as CKD stage 3, no ESRD occurred in this group within 10 years (Fig 2A). For those diagnosed at stage 4, 50% of individuals with annual decline rate > 3 ml/min 1.73m^2^ and between1 to 3 ml/min/1.73m^2^ enter ESRD within 3 years and 6 years, respectively (Fig 2B). For individuals with initial CKD stage 4 and annual decline rate <1 ml/min/1.73m^2^, more than 80% did not enter ESRD within 10 years. For those diagnosed initially at CKD stage 5, 50% of individuals with rapid decline will enter ESRD within one year and all of them enter ESRD within 4 years (Fig 2C). For CKD stage 5 individuals with annual decline rate between 1–3 ml/min/1.73m^2^, 50% of them enter ESRD around 2.5 years. Subjects with initial CKD stage 5 having average annual decline rate <1 ml/min/1.73m^2^ showed the best cumulative survival; however, most of them entered into ESRD status within 6 years.

## Discussion

CKD patients can have different disease course and declining trajectories of kidney function, which were not completely determined by the initial CKD stage; yet not all CKD patients entered into ESRD status in this 10-year CKD cohort. Measuring eGFR decline rate can help clinicians better predict outcomes of CKD. Absolute annual decline rate of eGFR is a useful therapeutic target to control CKD progression. Specifically, individuals with eGFR decline rate over 3 ml/min/1.73 m^2^/year require an intense focus on more integrated care delivery. Patients with age of CKD diagnosed < 60 year-old, diabetes and proteinuria tended to have a faster eGFR decline and thus the higher risk for ESRD.

There are different matrices to evaluate CKD progression: doubling of serum creatinine, a change of CKD stage, percentage change or absolute change of eGFR, or time to ESRD [11, 18–23]. Doubling of serum creatinine (equivalent to a 57% eGFR decline) is frequently used as the study endpoint [24]; however, it represents a relatively late event and is not sensitive enough to reflect early deterioration of renal function [21]. Some studies used percentage change of eGFR as an indicator of the progression of kidney function [11, 20]. A recent meta-analysis showed that eGFR decline quantified by percent change was strongly associated with the risk of ESRD and mortality [11]. The authors suggested a 30% reduction over 2 years could serve as an alternative endpoint [11]. In the present study, we demonstrated that annual eGFR decline rate simply based on the absolute difference over time is a useful predictor of disease progression and the risk of developing ESRD. According to previous studies, annual eGFR decline rate less than 1 ml/min /1.73 m^2^ /year was thought as a normal aging process [5, 19, 25–28]. The mean rate of eGFR decline of the Japanese general population was 0.36 mL/min/1.73 m^2^/year [28]. In the Baltimore Longitudinal Study on Aging, an average decline in creatinine clearance was 0.75 ml/min per year in American Adults [26]. The decline rate of eGFR greater than 1 ml/min /1.73 m^2^ /year among CKD patients is considered as disease progression. In our study, it was found that the risk for incident ESRD was 17% higher for each 1 ml/min/1.73 m^2^ increasing in annual decline rate.

There is little evidence that clearly specify the definition of “rapid CKD progression”. The 2002 K/DIGO guideline defined a fast CKD decline as ≥ 4 ml/min/1.73 m^2^/year; however, the 2012 K/DIGO guideline re-defined the rapid CKD progression as a sustained decline in eGFR of ≥5 ml/min/1.73 m^2^/year. However, these definitions are arbitrary and lacking in substantial evidence to support these definitions proposed. Results from a meta-analysis of 22 cohorts showed that eGFR decline slope greater than 3ml/min /1.73m^2^/year over the first 3years of CKD was significant associated with risk of ESRD (adjusted HR 1.73, 95% CI, 1.50 to 2.00)[29]. Using the data from the Alberta Kidney Disease Network (AKDN), among a cohort of 529,312 adults, the adjusted ESRD risks were 1.45, 1.53, 1.63, 1.90, 1.7 for the corresponding absolute annual rate of eGFR decline of -1, -2, -3, -4, and -5 ml/min/1.73 m^2^/year, respectively[5]. Another study including 15,465 patients with CKD demonstrated an increasing risk of mortality with increasing annual eGFR decline rate, even with decline rate 1 ml/min/1.73 m^2^/year[30]. In addition, the Cardiovascular Health Study showed that individuals with eGFR decline greater than 3 mL/min/1.73 m^2^ per year have an increased risk of cardiovascular events (heart failure, myocardial infarction, and peripheral arterial disease), cardiovascular and all-cause mortality[16, 17]. Our result supported a significant risk of ESRD associated with an annual decline rate steeper than 3 ml/min/1.73 m^2^ /year. Therefore, an annual eGFR decline rate of 3 ml/min/1.73 m2 /year may be specific enough to predict adverse CKD outcomes.

It is well known that certain pathologic conditions such as diabetes, proteinuria or hypertension would exacerbate eGFR decline rate [28, 31–34]. In MDRD study, six factors independently predicted a faster decline in GFR: greater urine protein excretion, polycystic kidney disease (PKD), lower serum transferrin, higher mean arterial pressure, black race, and lower serum HDL cholesterol[31]. In another study including 72,521 healthy Japanese, nine factors significantly influenced the slope of eGFR decline: baseline eGFR, age, gender, urinary protein, HbA1c, phosphorus, HDL-cholesterol, non-HDL-cholesterol, and BMI [27]. There are conflicting data about whether age at CKD diagnosis influences the rate of eGFR decline. The results from Baltimore Longitudinal Study showed that the rate of creatinine clearance decline increased with age [26]. A study from Japanese general population also found that eGFR decline was faster in older subjects with a lower baseline eGFR[28]. However, our study showed CKD patients with age greater than 60 years old were less likely to experience rapid CKD progression. Similar to our results, O’Hare et al. showed that older patients were associated with a slower decline in eGFR among patients with eGFR levels <45 ml/min per 1.73 m^2^ at baseline [35]. One possible explanation for O’Hare’s and our results is that the etiologies of impaired renal function among elderly are mainly due to physiologic aging rather than pathologic damage. Regarding gender differences in eGFR decline, a meta-analysis of studies among nondiabetic CKD found men had faster renal disease progression [36]. However, one Japan study showed that the rate of eGFR decline was slower in health men than in health women [27]. In our study, when we controlled for other factors, the annual eGFR decline rate was not significant different between men and women. Dyslipidemia was not associated with eGFR decline rate in the present study. Indeed, the role of dyslipidemia on CKD progression remains controversial [31, 37–39]. In the Modification of Diet in Renal Disease (MDRD) study, low HDL (high-density lipoprotein) is an independent risk factor for progression of renal disease [31]. However, in one large cohort of patients with CKD, total cholesterol, triglycerides, HDL, VLDL (very-low-density lipoproteins), LDL (low-density lipoprotein), apoA1 (Apolipoprotein A1), apoB (apolipoprotein B), and Lp(a) (Lipoprotein(a)) were not independently associated with the progression of kidney disease[37].

The associations between baseline eGFR and the rate of eGFR decline are inconsistent between studies. In MDRD study, the mean rate of GFR decline was not significantly related to baseline GFR[31]. However, among Japanese healthy adults, it was found the decline in eGFR was strongly correlated with baseline eGFR [27]. Also, a post hoc re-analysis of the JUPITER study showed that the magnitude of eGFR change was closely related to baseline eGFR, with greater reductions among subjects with eGFR**>** 60 mL/min/1.73m^2^ [40]. This observation was supported by our results showing that higher baseline eGFR was associated with higher annual decline rate (beta = 0.04, 95%CI: 0.02–0.06, p<0.001). Our study also revealed that it was the latest CKD stage, but not the baseline CKD stage, was associated with faster progression. Our findings indicated that eGFR decline rate is a better prognostic indicator than the baseline CKD stage. This implied that more effective therapies for slowing down the eGFR decline rate should be at the core of efforts to halt the CKD progression. The treatment plan should not simply determine by baseline CKD stage and individuals with a renal function decline rate over 3 ml/min/1.73 m^2^/year require intensive CKD care. Furthermore, researchers must be aware of the potential bias in interpreting clinical trials of kidney disease that only conduct randomization based on baseline CKD stage without taking eGFR decline rate into consideration.

The strength of this study was that we depicted the natural history of CKD progression from a large CKD cohort study with a long regular follow-up. Those who classified as early CKD (CKD stage 2) by current definition may not have significant eGFR decline and may not progress to ESRD. Our study had some limitations. First, we used the annual average eGFR decline rate, which assumed that a constant rate of eGFR decline over time; however, this model did not consider non-linear and time-varying pattern of the eGFR decline rate. However, this is a convenient and simple measurement to predict the progression of CKD during the course of follow-up. Second, residual confounding could not be completely eliminated, particularly because this study did not include the information of other renoprotective or nephrotoxic agents use in the analyses. Third, this is a single-center study in a Taiwanese population under Universal Health Care Coverage. This may diminish the generalizability of our study results to other centers and populations. Further studies are required to replicate our findings in other populations under different health care systems.

## Conclusions

Our study suggested that the annual eGFR decline rate performed well to predict CKD progression and ESRD events and the predictive performance may be better than the baseline CKD stage. Patients with CKD stage 2 or 3 may have a decline rate comparable to normal aging process and not all of them progressed to ESRD within a 10-year follow-up. Individuals with a renal function decline rate over 3 ml/min/1.73 m^2^/year require intensive CKD care. We believed our studies added evidence that the rates of eGFR decline are as important as, or more important than, a cross-section CKD staging as a predictor of CKD progression.
